# Microbiological findings in prepubertal and pubertal girls with vulvovaginitis

**DOI:** 10.1007/s00431-022-04631-4

**Published:** 2022-09-26

**Authors:** Stavroula Baka, Stiliani Demeridou, George Kaparos, Konstantinos Tsoutsouras, Sotirios Touloumakos, Maria Dagre, Sofia Meretaki, Anthia Chasiakou, Vasiliki Koumaki, Athanasios Tsakris

**Affiliations:** 1 Department of Biopathology, Aretaieion University Hospital, Medical School, National and Kapodistrian University of Athens, 76, Vasilisis Sofias Avenue, 11528 Athens, Greece; 2 Department of Microbiology, Medical School, National and Kapodistrian University of Athens, 75, Mikras Asias Street, Athens, 11527 Greece

**Keywords:** Vulvovaginitis, Puberty, Pathogens, Girls

## Abstract

Vulvovaginitis is a common and challenging gynaecological problem in prepubertal and pubertal girls. Such an infection, owing to a wide range of aetiologies, if not responding to hygienic measures, needs further investigation through vaginal cultures, since treatment should be tailored accordingly. This study aimed to investigate the pathogens isolated in prepubertal and pubertal girls with signs and symptoms of vulvovaginitis. A total of 2314 symptomatic girls, 1094 prepubertal and 1220 pubertal, aged 2 to 16 years, were included. Vaginal samples were inoculated on specific culture plates followed by incubation in aerobic, anaerobic or CO_2_ atmosphere at 37 °C for 24 or 48 h, as appropriate. The identification of the isolated pathogens was carried out using Gram stain, conventional methods and the automated system VITEK 2 (BioMerieux, Marcy l’Etoile, France). Positive cultures were obtained from 587 (53.7%) of prepubertal girls and 926 (75.9%) of pubertal girls. A total of 613 and 984 pathogens were detected in prepubertal and pubertal subjects, respectively. Isolated bacteria included 40.1% and 22.8% Gram-positive cocci, 35.6% and 24.8% Gram-negative rods in the prepubertal and pubertal groups, respectively, with faecal pathogens being the most prevalent. Bacterial vaginosis was diagnosed in 22.8% of prepubertal and 37.9% of pubertal girls. *Candida* species were isolated mostly in the pubertal girls (14.5%).

*Conclusion*: Culture results should be evaluated with caution in children with vulvovaginitis. In the prepubertal girls, the most common isolated pathogens were opportunistic bacteria of faecal origin while girls in late puberty were more susceptible to bacterial vaginosis and vulvovaginal candidiasis.

**Table:** 

**What is Known:** *• Vulvovaginitis is the most frequent and challenging reason for referral to paediatric and adolescent gynaecology services.* *• Microbiological examination can prove to be a significant tool to help diagnosis although results should be evaluated with caution in children.*	
**What is New:** *• Significantly more positive vaginal cultures and pathogens were recorded in symptomatic pubertal girls compared to prepubertal children.* *• The prevalence of bacterial vaginosis was increased in both prepubertal and pubertal girls with vulvovaginitis although significantly more in girls at puberty.*

**What is Known:**

*• Vulvovaginitis is the most frequent and challenging reason for referral to paediatric and adolescent gynaecology services.*

*• Microbiological examination can prove to be a significant tool to help diagnosis although results should be evaluated with caution in children.*

**What is New:**

*• Significantly more positive vaginal cultures and pathogens were recorded in symptomatic pubertal girls compared to prepubertal children.*

*• The prevalence of bacterial vaginosis was increased in both prepubertal and pubertal girls with vulvovaginitis although significantly more in girls at puberty.*

## Introduction

Vulvovaginitis is recognized as a common complaint of the genital system in prepubertal and pubertal girls causing anxiety in both parents and children. Different physical, chemical, or infectious agents have been implicated as causes of this clinical entity [1]. Although the suspicion of sexual abuse must always be investigated and ruled out, previous data suggest that mostly in prepubertal girls, vulvovaginitis is usually nonspecific, caused by irritants, allergic reactions or dermatological conditions [2].

Before puberty, the lack of oestrogens and the usually neutral or alkaline vaginal pH with no or few lactobacilli, create favourable environment for infections [1, 3]. In contrast, puberty represents a significant transition period from childhood to adulthood. With its onset, lactobacilli are increasing and become the predominant part of the vaginal microbiome, and the pH turns acidic [4]. Apart from maintaining an acidic vaginal pH, lactobacilli have an important role by minimizing the risk of vaginal infections. Any disruption of the healthy vaginal environment can predispose the patient to vulvovaginitis. In the pubertal patient, infectious vulvovaginitis is more common, and sexually transmitted infections must also be considered and investigated [5].

The female anatomy, with the urethra and anal region in close proximity to the vagina, puts the genital tract at risk of infection, in particular when local hygiene is poor or inadequate. Moreover, oropharyngeal pathogens can easily reach the genital area through self-inoculation. Such an infection, if not responding to hygienic measures, needs further investigation since treatment should be tailored to each specific patient. Microbiological diagnosis, by means of microscopic evaluation and cultures of vulvovaginal samples, can prove an important tool to exclude or to identify pathogens implicated in vulvovaginal infections. However, the significance of the pathogens isolated from the vaginal cultures must be evaluated only after taking into consideration clinical information and possible risk factors, if any. We conducted this study to investigate the pathogens isolated in prepubertal and pubertal girls presenting to our hospital with signs and symptoms of vulvovaginitis.

## Materials and methods

This was a retrospective analysis of data from 2314 girls aged 2 to 16 years, who presented at the Outpatient Clinic for Pediatric and Adolescent Gynecology of Aretaieio University Hospital, with symptoms suspicious of vulvovaginitis (vaginal discharge, genital erythema, pruritus, pelvic pain, vulvodynia, foul odour or bleeding), between January 2009 and December 2020. We excluded all cases referring to recurrent infections, vaginal foreign body or sexual abuse, as well as all girls that were currently under antibiotic therapy or in the previous month.

Vaginal samples were collected by trained paediatric and adolescent gynaecologists using a sterile new-born suction catheter carefully inserted into the vagina and immediately sent to the microbiology laboratory. Wet mount and Gram stain preparations were examined microscopically to assess for the presence of leukocytes, epithelial cells, trichomonads, clue cells, hyphae, pseudohyphae or budding yeasts. For the diagnosis of bacterial vaginosis (BV), Nugent score was applied on the Gram-stained vaginal smears and the number of lactobacilli, *Gardnerella vaginalis* and small and/or curved Gram variable morphotypes were assessed [6]. A score > 6 was indicative of BV.

Furthermore, vaginal samples were inoculated directly onto 5% sheep blood agar, MacConkey agar, Mannitol salt agar, chocolate agar and Wilkins Chalgren agar (Mast group Ltd, Merseyside, UK) as well as Sabouraud dextrose agar and *Gardnerella* agar (Liofilchem, Roseto d.Abruzzi, Italy) plates, followed by incubation in aerobic, anaerobic or CO_2_ atmosphere at 37 °C for 24 or 48 h, as appropriate. All microorganisms classified as pathogens were grown as a pure isolate or as the clear dominant bacteria on the culture plates and were further identified by conventional methods, using morphological and biochemical characteristics, Gram stain, while the definitive identification was carried out using the automated system VITEK 2 (Biomerieux, Marcy l’Etoile, France).

In children with genital pruritus or anal itching, a Graham test for pinworms was performed in the early morning for 3 consecutive days, by pressing a Sellotape strip on the skin adjacent to the anus which was then removed and placed onto a slide to be examined for the presence of *Enterobius vermicularis* ova by microscopy.

Statistical analysis was performed using the SPSS for Windows version 25.0 (SPSS Inc., Chicago, IL, USA). Categorical variables were analysed using the chi-square test for the comparison of data between groups. Statistical significance was set at a *P* value of 0.05.

## Results

Covering a period of 12 years, 2314 paediatric patients presenting with symptomatic vulvovaginitis were included in this study. Cases were divided into 2 groups: 1094 girls at prepubertal status Tanner stage 1, with absence of secondary sexual characteristics, and 1220 pubertal girls at Tanner stage ≥ 2. The most common clinical symptoms at presentation were vaginal discharge, genital erythema and pruritus (Table 1). Symptoms were significantly more prevalent in pubertal girls, except for vulvodynia which was more frequent among prepubertal girls.

**Table 1:** Clinical symptoms displayed by the patients with vulvovaginitis

**Clinical symptoms**	**Prepubertal (*n* = 1094)**	**Pubertal (*n* = 1220)**	**Total (*n* = 2314)**	***P***
Vaginal discharge	911 (83.3%)	1083 (88.8%)	1994 (86.2%)	< 0.01
Genital erythema	729 (66.6%)	818 (67.0%)	1547 (66.9%)	NS
Genital pruritus	652 (59.6%)	804 (65.9%)	1456 (62.9%)	< 0.01
Pelvic pain	378 (34.6%)	669 (54.8%)	1047 (45.2%)	< 0.01
Foul odour	220 (20.1%)	465 (38.1%)	685 (29.6%)	< 0.01
Genital bleeding	108 (9.9%)	252 (20.7%)	360 (15.6%)	< 0.01
Vulvodynia	188 (17.2%)	170 (13.9%)	358 (15.5%)	< 0.05

We collected one vaginal sample from each patient; thus, 2314 samples were collected in total. Significantly more (*P* < 0.01) positive cultures were recorded in girls at puberty (926/1220, 75.9%) when compared to the results obtained in the group of girls before puberty (587/1094, 53.7%). An increased number of pathogens was detected in the vaginal samples of pubertal subjects compared to the prepubertal ones (984 vs. 613, *P* < 0.01) (Table 2).

**Table 2:** Pathogens isolated in prepubertal and pubertal girls with vulvovaginitis

**Pathogens**	**Prepubertal (*n* = 613)**	**Pubertal (*n* = 984)**	***P***
Gram-positive cocci	246 (40.1%)	224 (22.8%)	< 0.01
* Enterococcus faecalis*	88 (14.4%)	69 (7.0%)	< 0.01
* Streptococcus pyogenes*	59 (9.6%)	46 (4.7%)	< 0.01
* Staphylococcus aureus*	35 (5.7%)	47 (4.8%)	NS
* Streptococcus agalactiae*	41 (6.7%)	22 (2.2%)	< 0.01
* Enterococcus faecium*	16 (2.6%)	36 (3.7%)	NS
* Staphylococcus lugdunensis*	7 (1.1%)	4 (0.4%)	NS
Gram-negative rods	218 (35.6%)	244 (24.8%)	NS
* Escherichia coli*	87 (14.2%)	65 (6.6%)	< 0.01
* Klebsiella pneumoniae*	25 (4.1%)	41 (4.2%)	NS
* Proteus mirabilis*	27 (4.4%)	24 (2.5%)	< 0.05
* Proteus vulgaris*	12 (2.0%)	19 (1.9%)	NS
* Pseudomonas aeruginosa*	13 (2.1%)	17 (1.7%)	NS
* Klebsiella oxytoca*	9 (1.5%)	18 (1.8%)	NS
* Enterobacter aerogenes*	7 (1.1%)	16 (1.6%)	NS
* Morganella morganii*	7 (1.1%)	11 (1.1%)	NS
* Haemophilus influenzae*	12 (2.0%)	5 (0.5%)	< 0.01
* Moraxella catarrhalis*	6 (1.0%)	10 (1.0%)	NS
* Enterobacter cloacae*	6 (1.0%)	6 (0.6%)	NS
* Citrobacter freundii*	3 (0.5%)	8 (0.8%)	NS
* Stenotrophomonas maltophilia*	4 (0.6%)	4 (0.4%)	NS
Bacterial vaginosis–associated pathogens	140 (22.8%)	373 (37.9%)	< 0.01
Yeasts (*Candida* spp.)	9 (1.5%)	143 (14.5%)	< 0.01

Among the Gram-positive cocci and the Gram-negative rods isolated in this study, faecal pathogens were the most prevalent. An unexpected finding was the increased prevalence of BV in both our groups, although significantly increased in pubertal girls (*P* < 0.01). Finally, *Candida* species were isolated mostly in the pubertal girls (*P* < 0.01) (Table 2).

## Discussion

Vulvovaginitis is one of the most common gynaecological complaints in the paediatric population. The microbial flora in girls with clinical signs and symptoms of vulvovaginitis is variable. What is considered as normal vaginal flora at different age periods is still a matter of dispute, and different data can be found in the literature. The vaginal microbiome is complex, and the presence of potential pathogens does not necessarily imply that they are responsible for the infection [7–9]. As a result, vaginal cultures obtained from vulvovaginitis cases must be cautiously evaluated before deciding specific antibacterial treatment.

The common causes of vulvovaginitis in the prepubertal patient are different than those considered in the pubertal patient. When children present with vulvar itching, burning or soreness, the most common aetiology is non-specific vulvovaginitis, no pathogen can be isolated, and hygiene measures are recommended [5]. Furthermore, younger girls cannot express with accuracy their symptoms; thus, clinical examination should make the distinction between non-specific and infectious vulvovaginitis [1]. Much attention is required for girls with symptoms secondary to intravaginal foreign bodies or sexually transmitted infections, where the concern of the specialists must concentrate on the possibility of sexual abuse [1, 2]. In this study, although a specific pathogen was not isolated from all cases, the clinical symptoms of all our patients were characteristic for vulvovaginitis. As previously reported, the most common complaints detected in our patients were vaginal discharge, vulvar erythema and pruritus [10–12].

Pathogens can reach the genital area from the surrounding skin as well as the rectal and urethral area [1]. The results of the vaginal cultures obtained from the prepubertal girls differed from the ones sampled from the pubertal girls. In prepubertal girls, we isolated mostly Gram-positive cocci and Enterobacteriaceae as demonstrated in earlier studies [11, 13]. In contrast, vulvovaginal candidiasis and BV were commonly detected in adolescents, as previously published [2, 14–16]. To our knowledge, there are very few reports in the literature comprising such large series as presented in this study.

The most frequently isolated pathogens in symptomatic paediatric patients with vulvovaginitis include respiratory tract and enteric microorganisms [3]. *Haemophilus influenzae* is a common pathogen of the respiratory tract. Young children, in particular, have usually a higher incidence of upper respiratory tract infections and, by touching the nose and mouth with their hands, they can easily spread the pathogens to other anatomical regions, as is the genital area. Although previous studies have pointed out that *H. influenzae* type b was one of the most common pathogen isolated in girls with vulvovaginitis, the introduction of the respective vaccine against this pathogen resulted in the significant decrease of its prevalence, thus protecting against respiratory tract infections and, as a result, against vulvovaginitis [1, 8, 17, 18]. The cases that are still reported, even in the vaccinated populations, are probably caused mostly by non-encapsulated or untypeable strains of *H. influenzae*. The low rate detected in our study may be attributed to the implementation of the vaccine from 1995 in Greece, and this result is in agreement with reports from different countries where vaccination is available [13, 19, 20]. In contrast, a recent report identified *H. influenzae* as the second most common pathogen in preschool-aged girls with a prevalence of 27.2% while McGreal and Wood reported it as the third most common pathogen with a prevalence of 10% in prepubertal girls [12, 21].

*Streptococcus pyogenes* (group A beta-haemolytic *Streptococcus*) is an important human pathogen responsible for different infections in the human body. Often has been reported as the most prevalent or one of the commonest causes of vulvovaginitis in girls [8, 12, 13, 19, 20, 22]. It is a common pathogen for the upper respiratory tract, and thus, children who are colonized with *S. pyogenes* are at increased risk of streptococcal vulvovaginitis since this pathogen can be easily transferred in the genital area [23]. Although not the commonest pathogen in our study population, the prevalence of *S. pyogenes* was similar with recent reports [12, 20].

*Staphylococcus aureus* is a significant human pathogen. In children, it represents the leading cause of skin and soft tissue infections and subsequently may cause diverse and even invasive infections with significant morbidity and mortality. Although *S. aureus* nasal and skin colonization is associated with an increased risk of infection, its significance in vulvovaginitis remains controversial [20]. However, many reports in the literature have demonstrated that *S. aureus* is a significant pathogen in symptomatic patients with vulvovaginitis [2, 3, 5, 8, 19, 24]. In contrast, Jariene et al. isolated *S. aureus* in both study (9.6%) and control (7.1%) groups, in mixed culture and did not consider it as the main pathogen [20]. Nonetheless, Kim et al. isolated *S. aureus* in 15% of prepubertal girls with vulvovaginitis, while others, in accordance to our data, reported 5.8% prevalence [8, 12, 19].

*Streptococcus agalactiae* or group B beta-hemolytic *Streptococcus* frequently colonizes genitourinary and gastrointestinal tract as well as the oropharynx [25]. However, the implication of this pathogen during pregnancy for the health of the new-born is of utmost importance and is always a matter of concern in reproductive age women. *S. agalactiae* has been included as a common pathogen in symptomatic patients presenting with vulvovaginitis [5]. We isolated *S. agalactiae* from 6.7% and 2.2% of prepubertal and pubertal girls, respectively. Similar with our data, Randelovic et al. detected this pathogen in 7.0% of the prepubertal patients while others reported a much higher isolation rate of 12%, being the second most common cause of vulvovaginitis in their group of prepubertal girls [8, 21].

We report an increased prevalence of enteric uropathogens. The role of faecal pathogens in the pathogenesis of vulvovaginitis is still unclear and a matter of dispute among specialists, since these pathogens are considered contaminants in healthy control groups, as well [10, 13, 20, 24]. Interestingly, Gorbachinsky et al. reported a significant increase in uropathogenic bacteria (79%) in periurethral samples from girls with vulvovaginitis compared to girls without vulvovaginitis (18%) [26]. Randelovic et al. reported bacteria of faecal origin in 33.8% of symptomatic girls, in pure culture, mostly *Proteus mirabilis* (14.4%), *Enterococcus faecalis* (12.2%) and *Escherichia coli* (7.0%) [8]. Others have also isolated faecal pathogens in significant numbers [2, 19, 22]. Similarly to the previous reports, pathogens found in the faecal flora were a significant cause of vulvovaginitis in prepubertal and pubertal children in our study, probably due to poor hygiene regarding voiding and wiping habits.

In prepubertal girls, the vaginal pH is alkaline with a hypoestrogenic milieu which allows the overgrowth of different microorganisms often of enteric or oropharyngeal origin [2, 3, 9]. The frequent isolation of enteric pathogens in vaginal samples could be explained by the anatomical proximity of the vulva and anus, poor hygiene or hygiene habits that help colonization. Children have often poor hygiene in the anogenital region, do not wash their hands regularly and can easily transfer pathogens from the oropharynx to genital area. On the other hand, pubertal girls, although more resistant to infections compared to prepubertal children, due to an increased microbial flora, can still be prone to infections due to enteric pathogens. Many cases of vulvovaginitis will improve and resolve by implementing careful hygienic measures and improved perineal hygiene [24]. However, it has been suggested that symptomatic paediatric patients must be treated accordingly since gastrointestinal microorganisms are characterized by increased pathogenicity [5].

We diagnosed BV in significant percentages in both groups studied. BV has been rarely reported in the literature as a cause of vulvovaginitis in girls, and it was not associated with sexual activity [13, 27]. In a normal prepubertal vagina, lactobacilli are lacking and, along with different aerobic species, anaerobic bacteria can also be found [2, 7, 9, 15]. McGreal and Wood reported increased anaerobes (51%) in prepubertal girls [21]. However, in pubertal girls, the most common causes of infectious vulvovaginitis are BV, *Candida* species and *Trichomonas vaginalis* [2, 15]. Unfortunately, their exact prevalence is unknown since these infections are not reportable. In these girls where vaginal flora is enriched by lactobacilli, *G. vaginalis* and different anaerobes can be isolated even without any evidence of sexual transmission [1, 14, 21]. Huppert et al. reported a BV prevalence of 23% in adolescents 14 to 19 years old while Xu et al. reported an incidence of BV and intermediate type of BV of 25.7% and 19.3%, respectively, in girls in late puberty, stating that these girls are more susceptible to BV and that more attention should be paid to menstrual hygiene [16, 28]. Vaca et al. reported a 31.5% BV prevalence in adolescent girls in Ecuador, suggesting as possible factors for the increased prevalence, genetic, behavioural and environmental causes [14]. However, due to a higher rate of sexual activity in adolescents, one must also consider sexually transmitted infections, as well [5]. In fact, it has been documented that BV facilitates the acquisition of sexually transmitted infections [14].

BV differs from the rest of vaginal infections because usually, inflammation is not present. It is characterized by a shift in vaginal microflora with a significant decrease or even disappearance of lactobacilli and an increase in *G. vaginalis*, *Atopobium vaginae*, different anaerobic bacteria and a plethora of other microorganisms that cannot be cultured, and, as a result, their identification can be achieved only by molecular methods [29, 30]. Furthermore, it has been demonstrated that BV is not only a polymicrobial infection but in fact a more complex clinical entity since it is characterized by biofilm formation [30, 31], which could offer a possible explanation for the frequent recurrences observed [15]. The increased prevalence of the microorganisms associated with BV in both our groups, although significantly increased in the pubertal group, is a matter of concern for future implications in their reproductive potential since BV has been associated with pelvic inflammatory disease (PID) and thus, with subfertility [32].

Usually, vulvovaginitis due to *Candida* species, as an oestrogen-dependent condition, is more prevalent in pubertal girls and quite unusual in prepuberty [3, 4, 8, 10, 20]. Higher oestrogen levels during puberty together with higher glucose levels in the vaginal milieu and poor menstrual hygiene are predisposing factors for vaginal candidiasis [13, 16]. Undoubtedly, in the presence of risk factors such as recent systemic antibiotic treatment, immunosupression, diabetes mellitus or sexual abuse, the incidence may increase in both groups [33]. *Candida* species are members of the normal intestinal or skin flora. Most yeast infections are caused by *Candida albicans* while *non-albicans* species exhibit a decreased susceptibility to antimycotics, specifically to topical or oral azoles which are currently used as a first-line treatment [2]. In our study, *Candida* vulvovaginitis was diagnosed significantly more often in pubertal compared to prepubertal girls, the result of which is in agreement with previous work [13, 14, 16]. In contrast, Hu et al. [12] reported an increased prevalence of 22.3% in a large group of prepubertal girls while Xu et al. [16] isolated *Candida* in 29.4% of girls in late puberty.

There are some limitations to the present study. We did not include a healthy asymptomatic control group to compare findings. However, this would have been very difficult to attempt since it is not easy to obtain permission from parents or guardians to collect vaginal samples from asymptomatic virginal healthy girls. Also, we did not perform in parallel nasopharyngeal cultures nor did we study the gastrointestinal flora of our patients. These cultures would have provided us with significant information regarding the pathogens that colonized our patients. Finally, the retrospective record review limited the possibility to collect and analyze treatment outcomes.

## Conclusions

Although vaginal cultures represent a significant tool for the correct identification of the pathogens responsible, their results should be evaluated with caution when deciding the cause of vulvovaginitis in children. In the prepubertal girls, the most common isolated pathogens were opportunistic bacteria of faecal origin while girls in late puberty were more susceptible to BV and vulvovaginal candidiasis. Combining a thorough history with an appropriate physical examination and a methodical laboratory evaluation should lead the clinician to correct diagnosis and targeted treatment. Nonetheless, general recommendations about personal hygiene should always be provided to symptomatic children of all ages. Vulvovaginitis is the most frequent reason for referral to the paediatric and adolescent gynaecology services, and, in order to ensure the reproductive health later in life, awareness of the pathogens often implicated is essential.

## Abbreviations

BV: Bacterial vaginosis
PID: Pelvic inflammatory disease

## References

[1] Dei M, Di Maggio F, Di Paolo G, Bruni V (2010). Vulvovaginitis in childhood. Best Pract Res Clin Obstet Gynecol.

[2] Zuckerman A, Romano M (2016). Clinical recommendation: vulvovaginitis. J Pediatr Adolesc Gynecol.

[3] Romano ME (2020). Prepubertal vulvovaginitis. Clin Obstet Gynecol.

[4] Joishy M, Ashtekar CS, Jain A, Gonsalves R (2005). Do we need to treat vulvovaginitis in prepubertal girls?. BMJ.

[5] Loveless M, Myint O (2018). Vulvovaginitis - presentation of more common problems in pediatric and adolescent gynecology. Best Pract Res Clin Obstet Gynaecol.

[6] Nugent RP, Krohn MA, Hillier SL (1991). Reliability of diagnosing bacterial vaginosis is improved by a standardized method of gram stain interpretation. J Clin Microbiol.

[7] Xiaoming W, Jing L, Yuchen P, Huili L, Miao Z, Jing S (2021). Characteristics of the vaginal microbiomes in prepubertal girls with and without vulvovaginitis. Eur J Clin Microbiol Infect Dis.

[8] Randelovic G, Mladenovic V, Ristic L, Otasevic S, Brankovic S, Mladenovic-Antic S, Bogdanovic M, Bogdanovic D (2012). Microbiological aspects of vulvovaginitis in prepubertal girls. Eur J Pediatr.

[9] Verstraelen H, Vieira-Baptista P, De Seta F, Ventolini G, Lonnee-Hoffmann R, Lev-Sagie A (2022). The vaginal microbiome: I. Research development, lexicon, defining “normal” and the dynamics throughout women’s lives. J Low Genit Tract Dis.

[10] Cemek F, Odabaș D, Şenel Ü, Kocaman AT (2016). Personal hygiene and vulvovaginitis in prepubertal children. J Pediatr Adolesc Gynecol.

[11] Beyitler İ, Kavukcu S (2017). Clinical presentation, diagnosis and treatment of vulvovaginitis in girls: a current approach and review of the literature. World J Pediatr.

[12] Hu BF, Hua CZ, Sun LY, Fang C, Zhou MM (2021). Microbiological findings of symptomatic vulvovaginitis in Chinese prepubertal girls. J Pediatr Adolesc Gynecol.

[13] Yilmaz AE, Celik N, Soylu G, Donmez A, Yuksel C (2012). Comparison of clinical and microbiological features of vulvovaginitis in prepubertal and pubertal girls. J Formos Med Assoc.

[14] Vaca M, Guadalupe I, Erazo S, Tinizaray K, Chico ME, Cooper PJ, Hay P (2010). High prevalence of bacterial vaginosis in adolescent girls in a tropical area of Ecuador. BJOG.

[15] Itriyeva K (2020). Evaluation of vulvovaginitis in the adolescent patient. Curr Probl Pediatr Adolesc Health Care.

[16] Xu L, Hu Z, Yu F, Tang Y (2020). Analysis of characteristics of vulvo-vaginal infections in 14- to 18-year-old girls in late puberty. J Int Med Res.

[17] Tartaglia E, Giugliano B, Ucciferri C, Giannattasio A, Giuliano P, Iannaccone VL (2013). Vulvovaginitis in prepubertal girls: new way of administering old drugs. J Pediatr Adolesc Gynecol.

[18] Li JP, Hua CZ, Sun LY, Wang HJ, Chen ZM, Shang SQ (2017). Epidemiological features and antibiotic resistance patterns of *Haemophilus influenzae* originating from respiratory tract and vaginal specimens in pediatric patients. J Pediatr Adolesc Gynecol.

[19] Kim H, Chai SM, Ahn EH, Lee MH (2016). Clinical and microbiological characteristics of vulvovaginitis in Korean prepubertal girls, 2009–2014: a single center experience. Obstet Gynecol Sci.

[20] Jarienė K, Drejerienė E, Jaras A, Kabašinskienė A, Čelkienė I, Urbonavičienė N (2019). Clinical and microbiological findings of vulvovaginitis in prepubertal girls. J Pediatr Adolesc Gynecol.

[21] McGreal S, Wood P (2013). Recurrent vaginal discharge in children. J Pediatr Adolesc Gynecol.

[22] Bumbulienè Ž, Venclavičiutè K, Ramašauskaite D, Arlauskiene A, Bumbul E, Drasutiene G (2014). Microbiological findings of vulvovaginitis in prepubertal girls. Postgrad Med J.

[23] Hansen MT, Sanchez VT, Eyster K, Hansen KA (2007). *Streptococcus pyogenes* pharyngeal colonization resulting in recurrent, prepubertal vulvovaginitis. J Pediatr Adolesc Gynecol.

[24] Jasper J (2009). Vulvovaginitis in the prepubertal child. Clin Ped Emerg Med.

[25] Armistead B, Oler E, Adams Waldorf K, Rajagopal L (2019). The double life of group B streptococcus: asymptomatic colonizer and potent pathogen. J Mol Biol.

[26] Gorbachinsky I, Sherertz R, Russell G, Krane L, Hodges S (2014). Altered perineal microbiome is associated with vulvovaginitis and urinary tract infection in preadolescent girls. Ther Adv Urology.

[27] Bechtel K (2010). Sexual abuse and sexually transmitted infections in children and adolescents. Curr Opin Pediatr.

[28] Huppert JS, Hesse EA, Bernard MC, Bates JR, Gaydos CA, Kahn JA (2012). Accuracy and trust of self-testing for bacterial vaginosis. J Adolesc Health.

[29] Srinivasan S, Fredricks DN (2008). The human vaginal bacterial biota and bacterial vaginosis. Interdiscip Perspect Infect Dis.

[30] Lev-Sagie A, De Seta F, Verstraelen H, Ventolini G, Lonnee-Hoffmann R, Vieira-Baptista P (2022). The vaginal microbiome: II. Vaginal dysbiotic conditions. J Low Genit Tract Dis.

[31] Verstraelen H, Swidsinski A (2013). The biofilm in bacterial vaginosis: implications for epidemiology, diagnosis and treatment. Curr Opin Infect Dis.

[32] Ravel J, Moreno I, Simon C (2021). Bacterial vaginosis and its association with infertility, endometriosis, and pelvic inflammatory disease. Am J Obstet Gynecol.

[33] Banerjee K, Curtis E, de San LC, Graham JC (2004). Low prevalence of genital candidiasis in children. Eur J Clin Microbiol Infect Dis.

